# Water, Sanitation, and Hygiene (WASH) Practices Among Households in Perambalur District: A Cross-Sectional Study

**DOI:** 10.7759/cureus.30115

**Published:** 2022-10-10

**Authors:** Tamilarasan Muniyapillai, Karthikeyan Kulothungan, Nawin J Vignesh, Rock B Dharmaraj, Neethu George

**Affiliations:** 1 Community Medicine, Dhanalakshmi Srinivasan Medical College and Hospital, Perambalur, IND

**Keywords:** diarrhea, safe drinking water, hygiene, sanitation, wash

## Abstract

Background

Sanitation, cleanliness, and access to clean water are essential for maintaining human health and well-being. Poor water, sanitation, and hygiene (WASH) practices are linked to diseases that lead to poor health outcomes, such as pneumonia and diarrhea, trachoma, infestations of soil-transmitted helminths, respiratory tract infections, and pulmonary tuberculosis. The aim of this study is to evaluate household WASH practices in the rural and urban field practice areas of the Dhanalakshmi Srinivasan Medical College and Hospital, Perambalur, India, and identify the factors that influence them.

Methodology

We conducted a five-month cross-sectional survey with 278 households in the rural and urban field practice areas of a tertiary care center in Perambalur district. We gathered data using the core questions developed by the World Health Organization-United Nations Children’s Fund (WHO-UNICEF) Joint Monitoring Programme (JMP), which is affiliated with UN-Water. Data analysts used a Pearson chi-square test to assess the relationship between WASH practices and several independent covariates, and we regarded P < 0.05 to be statistically significant.

Results

Among the 278 households, 118 respondents were from rural areas and 160 (57.7%) from urban areas. For drinking water, 31.4% of rural households used tube wells or boreholes, while 56.8% of those used public taps. In metropolitan areas, 25.6% of people used bottled water and 54.4% used water from tanker trucks. In terms of sanitation, 25.2% of households lacked a toilet and 72.7% used the flush/pour flush technique. Water, sanitation, and hygiene practices have been found to be associated with a population’s socioeconomic status and place of residence. The Pearson chi-square test revealed that the rural population has 99.1% more improved drinking water sources than the urban population, which was statistically significant (P < 0.001).

Conclusion

In rural areas, nearly 92% of households used improved sources of drinking water compared to only 55% of households in urban areas. On the other hand, just 47.5% of households in the rural area had improved sanitation compared to 95% of households in the urban area. Therefore, the Indian government should take steps to enhance urban drinking water quality and rural sanitation infrastructure.

## Introduction

One of the most often used and profound quotations is “health is hygiene” [1]. Good sanitation has a positive impact on all aspects of life, including health, nutrition, development, economy, dignity, and empowerment [2]. Infections such as cholera, typhoid, hepatitis A, and many other water-related diseases are more likely to spread when there are limited access to clean water, poor sanitation, and poor hygiene habits [3,4].

Water, sanitation, and hygiene (WASH) continues to be essential for maintaining public health. As a result, the sixth goal of the Sustainable Development Goal (SDG) seeks to ensure that everyone has access to safe drinking water and sanitary conditions. SDG target 6.1 aims to provide everyone with fair access to safe, affordable drinking water, while SDG target 6.2 aims to abolish open defecation and facilitate access to sanitation and hygiene services, both of which are based on the previously mentioned goals. The World Health Organization (WHO) estimates that unsafe WASH practices contributed to 8.29 million global fatalities and 49.8 million disability-adjusted life years in 2016, with a mortality rate of 11.7 deaths per 100,000 people. This is according to an updated evaluation of WHO statistics [5].

Poor cleanliness and hygiene practices account for 90% of diarrhea-related mortalities worldwide, which is significantly more than the combined mortality from HIV/AIDS and malaria [6]. Of these, Southeast Asian and African low- and middle-income nations have the greatest mortality rates associated with poor WASH practices, with 15.4 and 45.8 mortalities per 100,000 people, respectively [7]. According to the WHO statistics, 782 deaths per 1,000 people in India are attributed to WASH, and it also accounts for 7.5% of all mortality [8].

Although the majority of waterborne diseases can be successfully treated with medicines, the rise in antibiotic resistance has increased pressure on public health professionals, the pharmaceutical sector, and legislators. Interventions focused on reducing the percentage of people with limited access to clean drinking water can help us achieve sustainable development. These initiatives could result in significant economic gains for the country [8,9].

India has made rapid progress in ending open defecation across the country, which significantly impacts improving water, sanitation, and hygiene (WASH). The Swachh Bharat Mission, the Jal Jeevan Mission, and WASH in Schools (including preschools known as “Anganwadis”) are a few of the government of India’s key initiatives that the United Nations Children’s Fund (UNICEF) supports [10].

The Swachh Bharat Abhiyan, notably in metropolitan India, has considerably expanded the availability of resources for sanitation, hygiene, and safe drinking water. The availability of basic safe water increased from 94.7% to 96.3% between 2000 and 2017, the availability of basic sanitation facilities increased from 49.3% to 72%, and since 2012, roughly 80% of the urban population has been served by basic hygiene services, according to data published by the Joint Monitoring Programme (JMP) for water supply, sanitation, and hygiene in December 2019 [11]. The Swachh Bharat Abhiyan has made WASH services more accessible, yet India’s sanitation issues persist [12].

In order to get better results, we still need more hygienic education and personal hygiene, although government organizations may assist with the infrastructure required to improve the level of sanitation in developing countries [13,14]. By strengthening the standards for cleanliness, hygiene, and water usage, they can efficiently manage many communicable diseases [15,16]. In India, the WHO has been facilitating support to important players in research, capacity-building, and the promotion of best practices as well as the improvement of drinking water quality. In the cities of Hyderabad, Nagpur, Bengaluru, Chandigarh, and Surat, a water safety plan has been supported as a pilot project [17].

Regulations and infrastructure growth will close the knowledge and practice gap in sanitation and drinking water. To successfully lessen the effects of poor water and sanitation practices, it is essential to understand the current situation and how current initiatives are affecting urban and rural areas. In addition, UNICEF supports the planning and implementation of district-wide WASH interventions as well as the integration of behavior modification into state and national regulations and costed plans for WASH in healthcare facilities.

In order to aid underperforming states and districts, UNICEF works in 16 states and 192 districts, provides technical assistance to the government, supports alternative service delivery methods, and mobilizes public institutions and partners, including the corporate sector, around WASH programs [10]. Using the WHO and UNICEF WASH questionnaire, the project aims to evaluate the prevalence of improved drinking water and sanitation hygiene in field practice areas of the Dhanalakshmi Srinivasan Medical College and Hospital and determine the impact of socioeconomic level on WASH practices.

## Materials and methods

Study design and period

In the urban and rural field practice area of the tertiary care center in Perambalur, Tamil Nadu, India, we carried out an analytical cross-sectional study in rural and urban households for five months, from April 2022 to August 2022.

Study site

According to 2019 data from the rural and urban health center, there are around 8,690 rural and 9,090 urban households present in the urban and rural field practice area of the tertiary care center in Perambalur, Tamil Nadu.

Sample size calculation

Aneesh noted in his review that 97.4% of families in urban India had improved sources of drinking water compared to 94.6% of homes in rural India [18]. Considering the improved sources of drinking water in rural areas (P = 94.6%, absolute error (d) of 3%, and 95% confidence interval (CI)), the estimated minimum sample size was 241 using the equation z^2^*p* (1-p)/d^2^.

Sampling method

The field training area of the tertiary care center includes 14 hamlets and 18 villages. With the help of a straightforward random sampling technique, we selected five villages and five hamlets. Using the population proportion to size technique, the study had 278 participants, of whom 118 were from rural areas and 160 were from urban areas.

Exclusion criteria

We have excluded the houses that refuse to take part in the study. During the study, we did not include any households that were still locked after three visits.

Ethical clearance

Before the study began, we obtained an ethical clearance certificate; the reference number was IECHS/ IRCHS/ No.164.B. All subjects gave written informed consent before being enrolled in the study.

Data collection

We performed data collection through direct house-to-house visits. The participants were the household’s housewives. We enlisted the head of the family as participants in place of housewives. We evaluated the socioeconomic status of participants using a modified BG Prasad scale. The participants provided information about their drinking water quality, sanitation, and hygiene routines.

**Figure 1:**
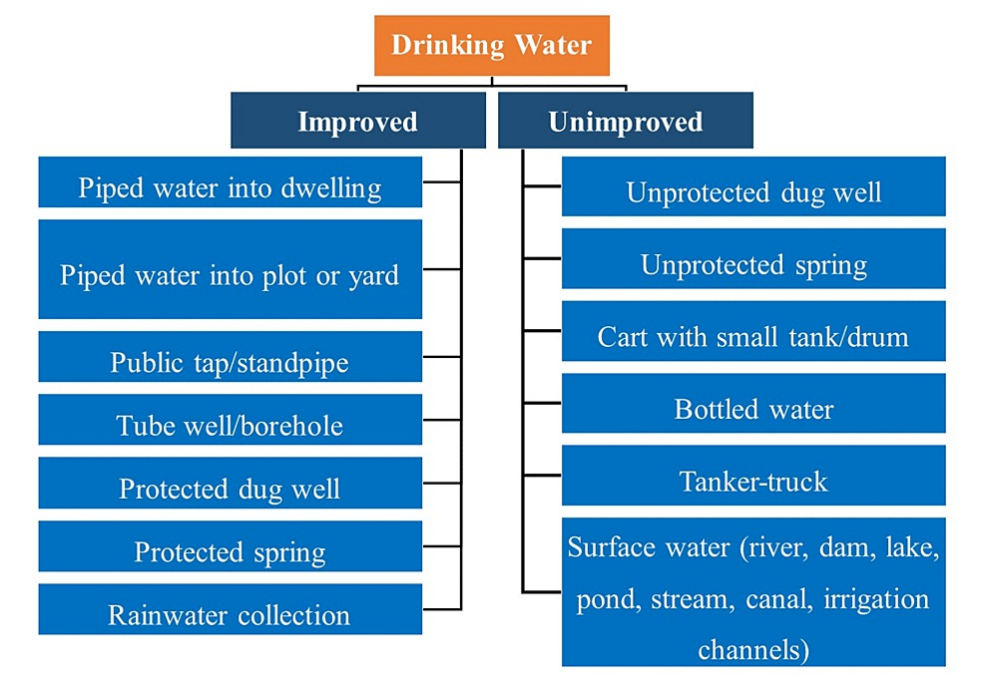
Improved and unimproved sources of drinking water

**Figure 2:**
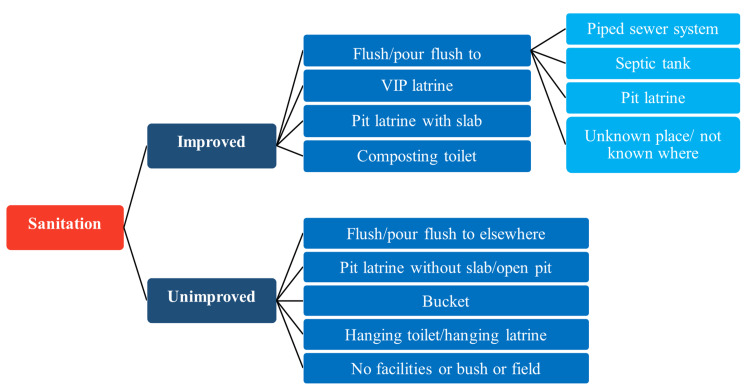
Improved and unimproved sanitation facilities

For the assessment of water and sanitation hygiene practices, we gathered data using the core questions developed by the WHO-UNICEF Joint Monitoring Programme (JMP), which is affiliated with UN-Water. The questionnaire had 10 questions in total. The inquiries were related to the supply of drinking water, water purification techniques used at home, and latrine facilities. We then divided them into improved and unimproved categories. We have described improved and unimproved drinking water sources and sanitation practices as reported by the WHO and UNICEF in Figure 1 and Figure 2.

Data analysis

Microsoft Excel (Microsoft Corp., Redmond, WA, USA) was used to enter all the gathered data. We then examined the distribution pattern of the data using SPSS version 20 (IBM SPSS Statistics, Armonk, NY, USA). Frequency and percentage were used to express descriptive variables. P < 0.05 was regarded as statistically significant when using the Pearson chi-square test to determine the relationship between the drinking water source and sanitation (dependent variable) and several independent variables (socioeconomic status and place of residence).

## Results

In the present study, 278 households were chosen and studied, of which 118 (42.4%) were from rural areas and 160 (57.6%) were from urban areas. Most of the urban population belonged to lower socioeconomic status (68.13%), whereas the rural population predominantly belonged to middle-income groups (63.56%) (Table 1).

**Table 1:** Socioeconomic status distribution in rural and urban areas

Residence	Socioeconomic status (%)			Total (%)
	Lower	Middle	Upper	
Rural areas	21.19	63.56	15.25	100
Urban areas	68.13	31.87	0	100

A cart with a small tank or drum served as the primary source of drinking water in urban households (54.38%), followed by bottled water (25.63%), piped water into the home (10.62%), and tube wells or boreholes (9.38%). In contrast, in rural areas, more than half of households (56.78%) used public taps or standpipes, while 31.36% used tube wells or boreholes. Approximately 68.71% of the premises had water (Table 2).

**Table 2:** Drinking water distribution in rural and urban areas

Drinking water	Urban (n = 160)	Rural (n = 118)	Total (N = 278)
Piped water into dwelling	17 (10.62%)	7 (5.93%)	24 (8.63%)
Piped water to yard/plot	-	6 (5.08%)	6 (2.16%)
Public tap/standpipe	-	67 (56.78%)	67 (24.10%)
Tube well/borehole	15 (9.38%)	37 (31.36%)	52 (18.71%)
Unprotected dug well	-	1 (0.85%)	1 (0.4%)
Cart with small tank/drum	87 (54.38%)	-	87 (31.29%)
Tanker truck provided	-	-	-
Bottled water	41 (25.63%)	-	41 (14.75%)
Total	160 (100%)	118 (100%)	278 (100%)

In urban areas, 45% of households had flush toilets connected to the sewerage system, and 46% had flush toilets connected to septic tanks. Only 5% of households practiced open defecation and had no toilets. However, just 45.76% of rural families had a flush toilet linked to the septic tank, and more than half of rural households (52.54%) practiced open defecation (Table 3).

**Table 3:** Sanitation distribution in rural and urban areas

Sanitation	Urban (n = 160)	Rural (n = 118)	Total (N = 278)
Flush/pour flush to a piped sewer system	41 (25.63%)	0	41 (14.75%)
Flush/pour flush to a piped sewer system (shared)	31 (19.36%)	0	31 (11.15%)
Flush/pour flush to the septic tank	61 (38.13%)	54 (45.76%)	115 (41.37%)
Flush/pour flush to the septic tank (shared)	13 (8.13%)	0	13 (4.68%)
Flush/pour flush to a pit	0	0	0
Flush/pour flush to a pit (shared)	0	0	0
Flush/pour flush to an unknown place	0	0	0
Flush/pour flush to an unknown place (shared)	0	0	0
No facilities/bush/field	8 (5%)	62 (52.54%)	70 (25.18%)
Other (shared)	6 (3.75%)	2 (1.69%)	8 (2.88%)
Total	160 (100%)	118 (100%)	278 (100%)

Water, sanitation, and hygiene practices have been found to be associated with a population’s socioeconomic status and place of residence. When compared to the urban population, the rural population had a larger percentage of improved drinking water sources (99.1%), which was statistically significant (P < 0.001) using a Pearson chi-square test. When compared to the rural population, we found that urban residents (91.3%) had better sanitation practices, which is statistically significant (P = 0.03). Higher socioeconomic groups had a higher percentage of improved drinking water sources (94.4%) compared to lower socioeconomic groups, which was statistically significant (P < 0.001) using a Pearson chi-square test. We discovered that lower socioeconomic groups (91.3%) had improved sanitation habits as compared to higher socioeconomic groups, which is statistically significant (P = 0.02) (Table 4).

**Table 4:** Influence of place of residence and socioeconomic status on drinking water and sanitation practices

Characteristics		Drinking water			Sanitation		
		Improved	Unimproved	P value	Improved	Unimproved	P value
Place of residence	Rural	117 (99.1%)	1 (0.8%)	0.0001	56 (47.5%)	62 (52.5%)	0.03
	Urban	73 (45.6%)	87 (54.4%)		146 (91.3%)	14 (8.8%)	
Socioeconomic status	Lower	62 (46.3%)	72 (53.7%)	0.0001	117 (87.3%)	17 (12.7%)	0.02
	Middle	111 (88.1%)	15 (11.9%)		84 (66.7%)	42 (33.3%)	
	Upper	17 (94.4%)	1 (5.6%)		1 (5.6%)	17 (94.4%)	

## Discussion

This study estimates the prevalence of WASH practices in South India as per the WHO-UNICEF Joint Monitoring Programme (JMP) for water supply and sanitation and determines their relationship with socioeconomic status and place of residence.

The current study found that 57.6% of people lived in urban areas and 42.4% of people were from rural areas. On the other hand, the 2011 census shows that 68.84% of people reside in rural areas compared to 31.16% who live in urban areas [19]. There are fewer cities in India, despite the fact that more people travel there in search of employment and a way to make a livelihood. The population of villages predominates when compared to a single rural area and a single city. The socioeconomic status, however, does not follow the same pattern, mostly because they move to cities in search of employment [20]. Therefore, lower socioeconomic status populations are more prevalent (68.13%) in urban regions, whereas middle socioeconomic status populations make up most of those who live in rural areas.

Only 45.6% of the urban population had access to improved sources of drinking water compared to over 99.1% of rural residents. According to JMP data for India, 84% of people in rural areas have access to the enhanced source compared to 96% of people in urban areas [11]. The reason for reduced access to improved sources of drinking water in urban areas might result from the socioeconomic status gap between rural and urban areas that are evident from this study observation.

Rural homes used a public tap or standpipe, whereas more than half of urban houses (54.3%) used a cart with a small tank or drum. According to a survey by Subbaraman et al. in urban slums, 99.6% of households obtain their drinking water from a public tap [21]. This may result from the current study being conducted among city dwellers while limiting the former study to urban slums. In the same study, they revealed that 84.9% of the population had a water supply on their property, while in the current study, only 68.71% of the people received water on their property.

Only 30.9% of the population in rural Tamil Nadu had access to a flush toilet that was connected to a septic tank or piped sewer system, according to research done there (Banda et al. [22]). About 74.2% of the population practiced open-air defecation [22]. Similar to the previous study, more than half of rural people (52.5%) practiced open defecation, whereas 91.3% of urban people had access to better sanitation facilities. It is possible that the similarities arise from the fact that, despite government efforts to encourage better sanitation practices, the habitual behavior of the populace resulted in worse sanitation practices. As a result, encouraging healthy habits from an early age is the only method to effectively promote practices related to water, sanitation, and hygiene.

Another significant finding in the current study was that we related WASH practices to residents’ places of living and socioeconomic position. This result was analogous to that of the Bangladeshi study by Raihan et al., which showed a relationship between residents’ places of residence and socioeconomic class and WASH behaviors [23].

Strength and limitation

The adoption of the WHO standard method for measuring hygiene practices is one of the study’s key strengths. For the current study area, there was no prior data on sanitation and hygiene habits, which would be helpful for implementing policies and programs in Perambalur district, Tamil Nadu, India.

Our study included a few restrictions. The participants voluntarily submitted all data. We did not examine their hygienic habits, which raises the possibility of self-reporting bias. We restricted the research to a single area, which is a low-performing state in India; we could not extrapolate the findings to other areas.

## Conclusions

Improved sanitation and drinking water sources aid in the prevention of childhood diarrhea and waterborne illnesses. Compared to half of the households in urban areas, the majority of households in rural areas used an improved supply of drinking water. Compared to the majority of households in urban areas, just half of the residences in rural areas have better sanitation. Despite having toilets, many rural residents opted to urinate outside. Habitual behaviors and sociocultural factors influence this practice. The construction of community and home toilets is a successful strategy for eradicating subpar sanitation habits, but health education is essential for preventing open defecation. The Swachh Bharat Abhiyan (Clean India Initiative), which aims to provide universal access to sanitation, is unlikely to be successful unless it focuses on many aspects of improving drinking water quality, sanitation, and hygiene practices.
